# Effects of Lewis number on the statistics of the invariants of the velocity gradient tensor and local flow topologies in turbulent premixed flames

**DOI:** 10.1098/rspa.2017.0706

**Published:** 2018-04-11

**Authors:** Daniel Wacks, Ilias Konstantinou, Nilanjan Chakraborty

**Affiliations:** 1 Department of Engineering, Durham University, Lower Mountjoy, South Road, Durham DH1 3LE, UK; 2 School of Mechanical and Systems Engineering, Newcastle University, Claremont Road, Newcastle upon Tyne NE1 7RU, UK

**Keywords:** premixed flame, turbulence, Lewis number, velocity gradient tensor, flow topology

## Abstract

The behaviours of the three invariants of the velocity gradient tensor and the resultant local flow topologies in turbulent premixed flames have been analysed using three-dimensional direct numerical simulation data for different values of the characteristic Lewis number ranging from 0.34 to 1.2. The results have been analysed to reveal the statistical behaviours of the invariants and the flow topologies conditional upon the reaction progress variable. The behaviours of the invariants have been explained in terms of the relative strengths of the thermal and mass diffusions, embodied by the influence of the Lewis number on turbulent premixed combustion. Similarly, the behaviours of the flow topologies have been explained in terms not only of the Lewis number but also of the likelihood of the occurrence of individual flow topologies in the different flame regions. Furthermore, the sensitivity of the joint probability density function of the second and third invariants and the joint probability density functions of the mean and Gaussian curvatures to the variation in Lewis number have similarly been examined. Finally, the dependences of the scalar--turbulence interaction term on augmented heat release and of the vortex-stretching term on flame-induced turbulence have been explained in terms of the Lewis number, flow topology and reaction progress variable.

## 1. Introduction

Recently, strict pollution control regulations have increased the need for low-emission premixed combustion, in which the reactants are homogeneously mixed prior to combustion. In premixed combustion, the maximum temperature attained when combustion is completed can be determined from the temperature and composition of the homogeneous mixture, because these quantities directly affect the combustion chemistry and the subsequent temperature field. As a result, pollutants such as NO*_x_* can be controlled with relative ease in premixed combustion by determining an optimal reactant mixture composition. Premixed combustion is used predominantly in spark ignition engines and industrial gas turbines and is now being developed for some modern aero-engines. In addition to the reduction of pollutant emissions, the emission of greenhouse gases such as CO_2_ also needs to be reduced in order to meet government regulations to tackle global warming. Several power generation techniques including non-conventional methods involving sustainable sources (e.g. solar power, wind power, tidal power) have been identified, but combustion is likely to remain a major contributor to industrial power generation for the foreseeable future because of existing expertise, infrastructure and the high reliability of conventional energy conversion methods. Hydrogen is often identified as a potential future fuel which would allow combustion with the complete elimination of greenhouse gas emission, but the chemistry of hydrogen is significantly different from that of hydrocarbon fuels [1], while the presence of lighter chemical species induces significant effects of the differential diffusion of heat and mass. The differential diffusion of heat and mass in premixed flames is often characterized in terms of the Lewis number *Le*, which is defined as the ratio of thermal diffusivity to mass diffusivity. Although every species in a combustion process has its own Lewis number, a characteristic Lewis number can be assigned to a given premixed combustion process in terms of the Lewis number *Le* of the deficient species [2,3] by heat release measurements [4] or by a linear combination of the mole fractions of the mixture constituents [5]. A number of analyses have demonstrated that the non-unity Lewis number has a significant influence on the burning rate and wrinkling of perturbed laminar flames and is also responsible for thermo-diffusive instability for *Le* < 1.0 (interested readers are referred to [6–8] and the references therein for an extensive review in this regard). Experimental investigations have indicated that the effects of the characteristic Lewis number do not disappear even for turbulent flames at high values of the turbulent Reynolds number [9,10]. It has been found that the rate of diffusion of fresh reactants into the reaction zone supersedes the rate at which heat is diffused out in the positively stretched zones for *Le* < 1.0 flames. This gives rise to the simultaneous presence of high reactant concentration and high temperature, and thus the burning rate and flame area generation are greater in the *Le* < 1.0 flames than in the unity Lewis number flames with similar turbulent flow conditions in the unburned reactants. Just the opposite mechanism gives rise to a reduced burning rate in the *Le* > 1.0 flames, in comparison with the corresponding unity Lewis number flame.

The augmentation of the burning rate with decreasing *Le* gives rise to a strengthening of flame normal acceleration and dilatation rate. This tendency is more prevalent in flames with *Le* < 1.0 due to thermo-diffusive instabilities [6–8]. The Lewis number dependences of the flame normal acceleration and dilatation rate have a significant influence on the turbulent kinetic energy and enstrophy transport through pressure gradient and baroclinic terms, respectively [11,12]. This leads to stronger flame-generated turbulence and enstrophy generation within the flame brush in the *Le* < 1.0 flames than in the unity Lewis number flame subjected to statistically similar unburned gas turbulence, and this tendency strengthens with decreasing Lewis number [11,12]. Strengthening of the flame normal acceleration eventually leads to counter-gradient transport of the turbulent kinetic energy, reaction progress variable, *c*, and its variance and dissipation rate with *Le* < 1.0 under similar turbulent conditions on the unburned gas side, for which gradient transport has been observed for flames with *Le* ≈ 1.0 [11,13–15]. The global Lewis number also significantly affects the alignment of the scalar gradient (i.e. the gradient of the reaction progress variable, ∇*c*) with local principal strain rates [16] through its influence on the flame normal acceleration and dilatation rate. It has been found that the reactive scalar gradient shows an increased tendency to align preferentially with the most extensive principal strain rate with decreasing Lewis number *Le*. This has been shown to have a significant influence on the normal strain rate contribution to the transport of the generalized flame surface density ($\text{FSD}=\bar{\left|\nabla c\right|}$, with the overbar indicating the Reynolds average/large eddy simulation (LES) filtering, as appropriate) and the scalar dissipation rate ($\text{SDR}=N_{c}=D\nabla c\cdot \nabla c$, with *D* being the progress variable diffusivity) [14,17]; the normal strain rate contribution is found to dissipate the FSD and SDR in flames with *Le* < 1.0 under similar turbulent conditions on the unburned gas side, for which the scalar gradient is created by the normal strain rate contribution in the FSD and SDR transport equations for flames with *Le* ≈ 1.0. The increased heat release with decreasing *Le* leads to strengthening of the dilatation rate and its contribution to the FSD and SDR transports. The contribution of the dilatation rate to the FSD and SDR acts to generate the FSD and SDR for all flames irrespective of *Le,* but this effect strengthens with decreasing *Le* [14,17].

The differential diffusion of heat and mass has a significant influence on the flame curvature (i.e. the curvature of a given *c*-isosurface, given by $\kappa_{m}=0.5\nabla \cdot (-\nabla c/|\nabla c|)$) dependences of the temperature and heat release rate in non-unity Lewis number flames [13,18,19]. This gives rise to a finite probability of finding super-adiabatic temperatures even under globally adiabatic conditions. These super-adiabatic hot spots in the *Le* < 1.0 cases play an important role in increasing the wall heat flux and quenching the distance for head-on quenching of turbulent premixed flames [20]. Furthermore, the aforementioned curvature dependence of the chemical reaction rate affects the local stretch rate dependence of the flame displacement speed *S*_d_ [19]. This, in turn, affects the statistical behaviours of the curvature and propagation terms of the FSD and SDR transport equation.

From the above discussion, it is evident that the Lewis number pervasively influences the heat, mass and fluid flow processes in turbulent premixed flames, and these influences are likely to have a significant influence on the underlying flow topology in turbulent premixed combustion.

**Figure 1.**
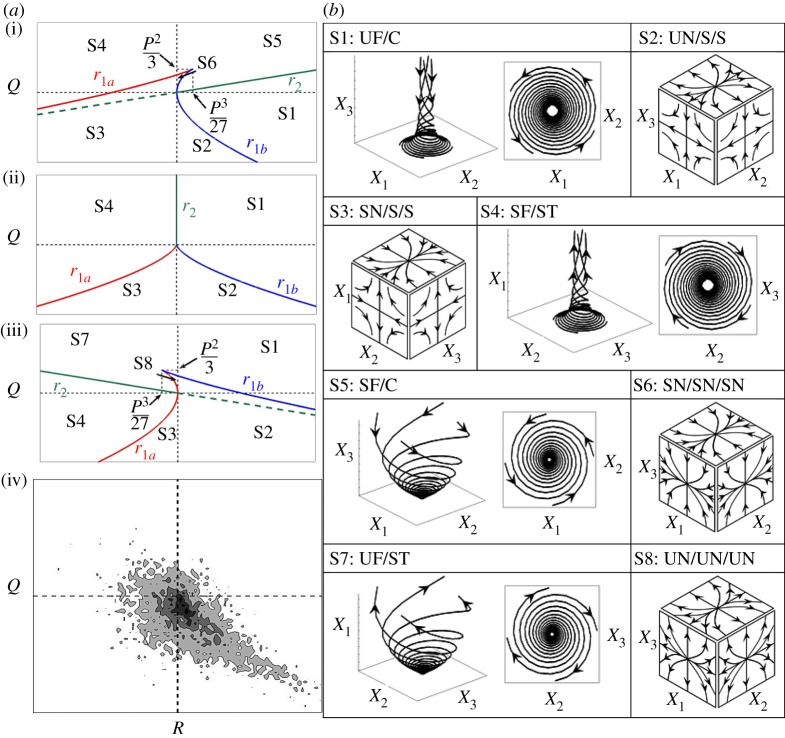
(*a*) Classification of the S1–S8 topologies in the *Q*−*R* plane for (i--iii) *P *> 0, *P *= 0 and *P *< 0*,* and (iv) the ‘teardrop’-shaped PDF(*R*,*Q*) lying in the *P *= 0 plane. The lines $r_{1a}$ (red), $r_{1b}$ (blue) and *r*_2_ (green) dividing the topologies are shown. Black dashed lines correspond to *Q *= 0 and *R *= 0. (*b*) Classification of the S1–S8 topologies: UF, unstable focus; UN, unstable node; SF, stable focus; SN, stable node; S, saddle; C, compressing; ST, stretching. (Reproduced with permission from Wacks & Chakraborty [23].)

Perry & Chong [21] and Chong *et al*. [22] classified all the possible flow topologies in terms of the invariants (*P*, *Q* and *R*) of the velocity gradient tensor with components given by ∂*u_i_*/∂*x_j_*, where *u_i_* is the *i*th component of the velocity vector. The topologies, denoted S1–S8, distinguish eight regions in the three-dimensional *P*–*Q*–*R* phase space, as shown schematically in figure 1. For incompressible flows, the first invariant P=−∇⋅u is exactly zero, such that incompressible flow topologies are dependent only on the second and third invariants (i.e. *Q* and *R*). Perry & Chong [21] and Soria *et al.* [24] concluded, based on their analyses, that the S4 topology most likely occurs for positive values of *Q*. Blackburn *et al*. [25] revealed that topologies S2 and S4 are dominant in the regions away from the wall. Chong *et al.* [26] and Chacin & Cantwell [27] revealed that the joint probability density function (PDF) between *Q* and *R* demonstrates a ‘teardrop’ structure (figure 1). In addition, Ooi *et al*. [28] indicated that the joint PDF between *Q* and *R* tends to show similar qualitative behaviour for a range of different incompressible turbulent flows, suggesting a degree of universality of small-scale turbulent motion in the *Q*–*R* plane. The ‘teardrop’ structure of the *Q*–*R* joint PDF for incompressible flows has been confirmed by experimental results [26,27]. Elsinga & Marusic [29] offered an explanation for the universal ‘teardrop’ shape of the *Q*−*R* joint PDF for incompressible flows. Tsinober [30] provided qualitative arguments for local flow properties for different topologies, and postulated that the enstrophy production is large in the S4 topology, whereas the strain rate production is concentrated in the S1 topology. Dopazo *et al.* [31] examined the interaction of flow topologies with passive scalar surface topologies quantified in terms of Gauss and mean curvatures (i.e. *κ*_g_ and *κ*_m_). Direct numerical simulations (DNSs) and experimental investigations revealed that the ‘teardrop’ structure of the *Q–R* joint PDF exists only in the fully turbulent region and not in the interface between the turbulent and non-turbulent regions [32,33]. All of these aforementioned analyses were carried out for incompressible fluids, but in compressible flows the first invariant of the strain rate tensor, *P*, plays a key role in addition to *Q* and *R*, and thus the three-dimensional *P–Q–R* space plays a pivotal role. Chen *et al.* [34] analysed the structure of a compressible wake in terms of *P*, *Q* and *R*. Sondergaarad *et al.* [35] also used the scatter plots of *P*, *Q* and *R* to analyse the local flow geometry of a turbulent shear flow. Maekawa *et al.* [36] demonstrated that the S2 and S4 topologies dominate the *Q–R* plane for decaying isotropic turbulence, which was subsequently investigated by Suman & Girimaji [37]. Wang & Lu [38] analysed topology distributions in the inner and outer layers in turbulent compressible boundary layers.

The flow topology in turbulent premixed flames was analysed for the first time by Tanahashi *et al.* [39] to distinguish between strain-dominated (i.e. *Q* < 0) and vorticity-dominated (i.e. *Q* > 0) regions. They also demonstrated that coherent structures can survive beyond the flame front. Grout *et al*. [40] analysed the flow topology of a reactive transverse fuel jet in cross flow and demonstrated that the regions of the highest heat release rates of the flame are associated with the S8 topology. Recently, Cifuentes *et al.* [41,42] demonstrated, using a simple chemistry DNS database of premixed turbulent flames with unity Lewis number, that the probability of finding the focal (nodal) flow topologies decreases (increases) across the flame front. Furthermore, Wacks & Chakraborty [23] analysed flow topology distributions in turbulent spray flames and demonstrated that the distribution of topologies within the spray flame has qualitative similarities to the previous findings by Cifuentes *et al.* [41] and Grout *et al.* [40]. However, the influence of *Le* on the turbulent flow topology distribution in premixed turbulent flames is yet to be analysed and this paper addresses this gap in the existing literature. This void is addressed by analysing a DNS database of statistically planar turbulent premixed flames with global Lewis number *Le* ranging from 0.34 to 1.2. The specific objectives of this paper are as follows:

(1) To indicate, understand and provide physical explanations for *Le* dependence of the flow topology distribution within turbulent premixed flames.
(2) To demonstrate the implications of the above results on scalar dissipation production by scalar gradient normal stretching (henceforth referred to as the scalar--turbulence interaction term following previous analyses [15,16,43,44]) and on the vortex-stretching term in the enstrophy transport equation.

As the flow topologies are associated with particular combinations of strain rate and vorticity distributions, they are likely to influence the statistical behaviours of the scalar--turbulence interaction and vortex-stretching terms because these quantities are determined by the local alignment of the principal strain rate and scalar gradient and vorticity, respectively. Moreover, the vortex-stretching mechanism is of pivotal importance to the energy cascade in turbulent flows [45] and it plays a leading-order role in enstrophy transport in turbulent premixed combustion even though some other physical mechanisms (e.g. baroclinic torque) might also play leading roles, alongside the vortex-stretching term [12].

The analysis in terms of the aforementioned objectives is expected to reveal the canonical flow configurations, which make dominant contributions to the scalar--turbulence interaction and vortex-stretching terms of the SDR and enstrophy transport equations, respectively, for different values of the global Lewis number. This, in turn, helps to design simplified configurations representing dominant flow topologies to gain further insight into the flame--turbulence interaction and vortex-stretching terms, and thus these configurations can be used for experiments and Reynolds-averaged Navier–Stokes (RANS) simulations and LES for obtaining fundamental physical understanding and combustion model validation for premixed combustion with different values of the characteristic Lewis number.

The rest of the paper will be organized as follows. The mathematical background and numerical implementation pertaining to this analysis are presented in the next section. This will be followed by the presentation of the results and their discussion. The main findings will be summarized and conclusions will be drawn in the final section of this paper.

## 2. Mathematical background and numerical implementation

In order to analyse the effects of the global Lewis number *Le* on individual flow topologies the chemical mechanism in this analysis is simplified by a single-step Arrhenius-type chemical reaction following several previous analyses [6–8,11–20,46–51]. In the context of simple chemistry, the species field in premixed turbulent flames is often represented by a reaction progress variable *c* in terms of the reactant mass fraction *Y_R_* in the following manner [6–8,11–20,46–51]:

2.1 $$c=\frac{Y_{R0}-Y_{R}}{Y_{R0}-Y_{R\infty }},$$

where the subscripts 0 and ∞ are used to refer to the values in the fully unburned reactants and fully burned products, respectively. According to the above equation, *c* increases monotonically from 0, in the unburned gas, to 1.0, in the fully burned products.

Following Perry & Chong [21] and Chong *et al*. [22], the local flow topologies can be characterized by the invariants of the velocity gradient tensor: *A_ij_* = ∂*u_i_*/∂*x_j_* = *S_ij_* + *W_ij_*, where *S_ij_* = 0.5(*A_ij_* + *A_ji_*) and *W_ij_* = 0.5(*A_ij_* − *A_ji_*) are the symmetric and anti-symmetric components, respectively. Three eigenvalues, *λ*_1_, *λ*_2_ and *λ*_3_, of *A_ij_* can be obtained from solutions of the characteristic equation *λ*^3^ + *Pλ*^2^ + *Qλ* + *R* = 0, where *P*, *Q*, *R* are the invariants of *A_ij_* [21,22],

2.2 $$\begin{array}{rl} & P=-(\lambda_{1}+\lambda_{2}+\lambda_{3}),\\ & Q=0.5(P^{2}-S_{ij}S_{ij})+0.5(W_{ij}W_{ij})=Q_{s}+Q_{w}\\ \text{and}\; & R=\frac{-P^{3}+3PQ-S_{ij}S_{jk}S_{ki}-3W_{ij}W_{jk}S_{ki}}{3}. \end{array}\}$$

The discriminant *D* = [27*R*^2^ + (4*P*^3^ − 18*PQ*)*R* + 4*Q*^3^ − *P*^2^*Q*^2^]/108 of *λ*^3^ + *Pλ*^2^ + *Qλ* + *R* = 0 divides the *P–Q–R* phase space into two regions: for *D* > 0 (*D* < 0), where *A_ij_* displays a focal (nodal) topology [21,22]. The *A_ij_* tensor exhibits one real eigenvalue and two complex conjugate eigenvalues for focal topologies, whereas *A_ij_* shows three real eigenvalues for nodal topologies. The surface *D* = 0 leads to two subsets *r*_1*a*_ and *r*_1*b*_ in *P–Q–R* phase space which are given by [21,22]: *r*_1*a*_ = *P*(*Q* − 2*P*^2^/9)/3 − 2(−3*Q* + *P*^2^)^3/2^/27 and *r*_1*b*_ = *P*(*Q* − 2*P*^2^/9)/3 + 2(−3*Q* + *P*^2^)^3/2^/27. In the region *D* > 0, *A_ij_* has purely imaginary eigenvalues on the surface *r*_2_, which are given by *R* = *PQ*. The surfaces *r*_1*a*_, *r*_1*b*_ and *r*_2_, where *r*_2_ is described by *PQ* − *R* = 0, divide the *P–Q–R* phase space into eight flow topologies, as shown in figure 1. Interested readers are referred to the appendix of [22] for further explanation.

The distribution of these flow topologies for different values of *Le* has been analysed here by using a three-dimensional DNS database of statistically planar turbulent premixed flames with global Lewis number *Le* ranging from 0.34 to 1.2. This database has been used several times in the existing literature [11–17] and thus only a brief discussion is provided here. The simulations have been conducted using a well-known DNS code SENGA [11–17], where the governing equations of mass, momentum, energy and reaction progress variable are solved in non-dimensional form. Interested readers are directed to appendix A, where the full transport equations are presented. The computational domain is taken to be a cube of size 24.1*δ*_th_ × 24.1*δ*_th_ × 24.1*δ*_th_ (where $\delta_{\mathrm{th}}=(T_{\mathrm{ad}}-T_{0})/\max{\left|\nabla T\right|}_{L}$ is the thermal flame thickness with *T*, *T*_0_ and *T*_ad_ being the instantaneous dimensional temperature, unburned gas temperature and adiabatic flame temperature, respectively), which is discretized using a uniform Cartesian mesh of 230 × 230 × 230. This grid spacing ensures at least 10 grid points, and more than two grid points within the thermal flame thickness *δ*_th_ and the Kolmogorov length scale *η*. The spatial discretization for the internal grid points is carried out using a 10th order central difference scheme, but the order of differentiation gradually drops to a second-order one-sided finite difference scheme at the non-periodic boundaries. The temporal advancement has been carried out using a third-order low-storage Runge–Kutta scheme (A. A. Wray 1990, unpublished data). The turbulent velocity components have been initialized by an incompressible homogeneous isotropic field using a pseudo-spectral [52] method. The reacting scalar field is initialized by the steady planar laminar flame solution and the turbulent velocity field is superimposed on top of it. The initial values of *u*′/*S*_L_ and *l*/*δ*_th_ are taken to be 7.5 and 2.45 for five different global Lewis numbers *Le* = 0.34, 0.6, 0.8, 1.0 and 1.2 for cases A--E, respectively, where *u*′ is the root mean square (RMS) velocity fluctuation and *l* is the integral length scale. For these values of *u*′/*S*_L_ and *l*/*δ*_th_, the Damköhler number *Da* = *S*_L_*l*/*u*′*δ*_th_ and Karlovitz number *Ka* = (*u*′/*S*_L_)^3/2^(*l*/*δ*_th_)^−1/2^ are 0.33 and 13.0, respectively, for all cases considered here, and the combustion takes place nominally in the thin reaction zones regime [53]. Furthermore, the heat release parameter *τ* = (*T*_ad_ − *T*_0_)/*T*_0_ = 4.5 has the same value in all cases. The unity Lewis number flames are analogous to the stoichiometric methane–air flame, whereas the Lewis number 0.34 case is representative of a lean hydrogen–air mixture [5,10,54]. The Lewis number 0.6 and 0.8 cases are representative of hydrogen-blended methane–air mixtures (e.g. 20% and 10% (by volume), respectively, hydrogen-blended methane–air flames with an overall equivalence ratio of 0.6) and the Lewis number 1.2 case is representative of a hydrocarbon–air mixture involving a hydrocarbon fuel which is heavier than methane (e.g. ethylene–air mixture with an equivalence ratio of 0.7) [5,10,54]. The simulations for decaying turbulence should be conducted for time *t*_sim_ = max(*t*_c_, *t*_f_), where *t*_c_ = *δ*_th_/*S*_L_ and *t*_f_ = *l*/*u*′ are the chemical time scale and initial eddy turn-over time. For all cases considered here, simulations have been conducted for *t*_sim_ = *t*_c_, which corresponds to 3.34*t*_f_. By that time, *u*′ has decayed by 50% and *l* has increased by a factor of 1.7. Moreover, both the turbulent kinetic energy and dissipation rate in the unburned gas ahead of the flame did not change rapidly with time when the statistics were extracted. Further information about the conditions under which the statistics are taken for this analysis can be found in [11–17].

## 3. Results and discussion

**Figure 2.**
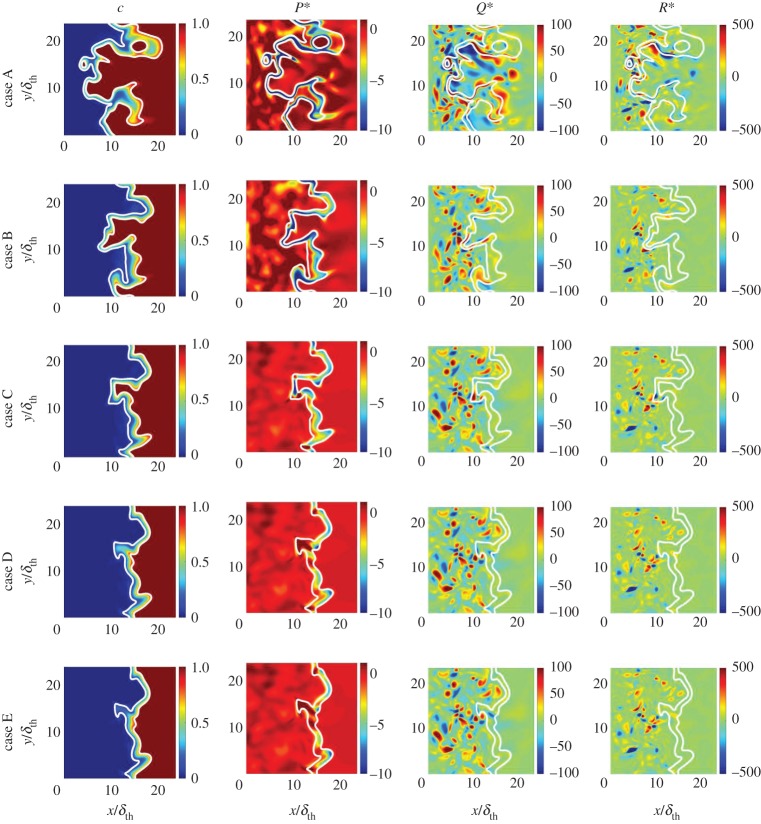
Selected regions of the instantaneous (column 1) reaction progress variable *c*, (column 2) normalized first invariant $P^{*}=P\times (\delta_{\mathrm{th}}/S_{L})$, (column 3) second invariant, Q* = *Q* × (*δ*_th_/*S*_L_)^2^, and (column 4) third invariant $R^{*}=R\times {\left(\delta_{\mathrm{th}}/S_{L}\right)}^{3}$ fields at the *x*–*y* mid-plane for (top to bottom) cases A–E. White contours show *c* = 0.1 (left) and 0.9 (right) isolines.

Figure 2 shows selected planes of the instantaneous reaction progress variable field, *c*, and the normalized first, second and third invariant fields: P* = *P* × (*δ*_th_/*S*_L_), Q* = *Q* × (*δ*_th_/*S*_L_)^2^ and R* = *R* × (*δ*_th_/*S*_L_)^3^, respectively. The data for these fields were extracted at the final time, *t*_sim_ = *t*_c_ = 3.34*t*_f_. Figure 2 also shows the location of the flame in the form of the selected contour lines of *c* = 0.1 and 0.9 (from left to right) superimposed on top of the reaction progress variable field. The *c*-contours show that there is a marked decrease in the level of flame wrinkling as the Lewis number increases from case A to case E (i.e. case A exhibits the greatest degree of wrinkling and case E the least). This is consistent with the increased burning rate and flame area generation associated with *Le* < 1.0 flames, due to the simultaneous presence of high reactant concentration and high temperature, in contrast to the reduced burning rate experienced by the *Le* > 1.0 flames. The augmentation of the burning rate in low Lewis number flames and its reduction in high Lewis number flames (in comparison with the unity Lewis number flame) is also evident from the P* fields. The first invariant, P=−∇⋅u, is the negative of the dilatation rate and, as such, strongly negative values of P* represent regions of high thermal expansion due to a locally high burning rate. Case A exhibits strong negative values of P* in the region of the flame front (compared with the location of the *c*-contour lines), and the magnitude of P* decreases with increasing *Le*. The high values of dilatation in the *Le* ≈ 1.0 flames (e.g. cases C–E) are to be found along the flame front in regions where the flame is concave towards the reactants, whereas in regions where the flame is convex towards the reactants the dilatation rate assumes more modest values. This behaviour follows from focusing (defocusing) of heat in the regions which are concave (convex) to the reactants. This tendency is particularly strong for *Le* > 1.0 cases because of stronger thermal diffusion out of the flame front than the reactant diffusion into it. This leads to the simultaneous occurrence of strong focusing of heat and weak defocusing of reactants in the regions which are concave to the unburned gases in the *Le* > 1.0 flames and thus the burning rate and thermal expansion effects (e.g. high magnitudes of the negative value of P*) are strong in these locations. This also gives rise to high burned gas temperatures in the regions which are concavely curved towards the unburned gas. Just the opposite mechanism leads to small values of burning rate, dilatation rate and burned gas temperature in the regions where the flame is convex to the reactants in the *Le* > 1.0 flames. In flames where *Le* < 1.0, the focusing of reactants at zones which are convex towards the reactants takes place at a faster rate than the thermal diffusion rate out of these reaction zones. This leads to high (in some cases even super-adiabatic) burned gas temperatures at the regions which are convex towards the reactants in the *Le* < 1.0 flames, and by the same token lower values of burned gas temperature in the regions which are concavely curved towards the unburned gas side. These high values of temperature at the convexly curved regions tend to induce high values of the dilatation rate in the *Le* < 1.0 flames in addition to the effects of focusing of heat at zones which are concavely curved towards the reactants. Thus, the high negative values of P* in the *Le* < 1.0 flames are not confined to zones which are concavely curved towards the reactants with *Le* ≈ 1.0 (e.g. cases C–E). Furthermore, temperature inhomogeneity is observed in the burned gas for non-unity Lewis number flames because of the inequality of the diffusion rates of species and heat (fig. 6 of [13] and fig. 1 of [14]), whereas the burned gas temperature remains equal to the adiabatic flame temperature for the unity Lewis number flames. The effect of this temperature inhomogeneity is relatively more prevalent for the *Le* < 1.0 flames than in the *Le* > 1.0 cases because the higher thermal diffusion rate in the *Le* > 1.0 flames tends to nullify thermal inhomogeneities in the burned gas. The temperature inhomogeneity in the burned gas gives rise to a considerable dilatation rate within the burned gas beyond the flame and this tendency strengthens with decreasing Lewis number. It is worth noting that the extent of flame wrinkling increases with decreasing *Le* and thus the flame wrinkles out of the plane shown in figure 1 can lead to significant magnitudes of P=−∇⋅u, which is reflected in the non-zero values of P* on the unburned gas side in cases A and B.

It can be seen from figure 2 that the distribution of the second invariant, Q*, appears similar both qualitatively and quantitatively in cases B–E, such that non-negligible values occur mostly in the unburned gas region which is composed of alternating small areas of positive and negative values. The magnitudes assumed by Q* in the burned gas region remain negligible. In case A, the non-negligible Q*-distribution penetrates the flame front to enter the burned side and consists of larger areas of highly positive and highly negative values compared with cases B–E. A similar scattered distribution is apparent for the third invariant, R*, in cases B–E in the unburned gas region. The distribution is once again stronger (more highly positive and highly negative) in case A and can be seen to penetrate the burned gas region. The scattered distribution evident in the unburned gas regions of both instantaneous Q* and R* fields arises because of the nature of the distributions and relative magnitudes of *S_ij_S_ij_* and *W_ij_W_ij_*, and a qualitatively similar behaviour was observed in previous analyses [23].

Although it is evident from equation (2.2) that the strain rate, vorticity and dilatation rate all contribute towards the value of *Q*, it is clear from figure 2 that the magnitude of *P*^2^ remains smaller than that of *Q* at most locations in the flow field and that these quantities are only comparable within the reaction zone. Since the aforementioned scattered distributions in cases B–E lie mainly in the unburned gas regions where *P* ≈ 0 (for low Mach number flows such as the ones considered in this study), we may approximate *Q*_S_ ≈ −0.5*S_ij_S_ij_*, which is always negative. Therefore outside the flame the sign of *Q* is indicative of vorticity-dominated regions (*Q* > 0) and strain-dominated regions (*Q* < 0). The aforementioned alternating positive and negative regions of Q* which can be seen in figure 2 show that both vorticity- and strain-dominated regions exist in all cases in the unburned gas region, and, in case A, also in the burned gas region.

The third invariant, *R* (equation (2.2)), may be rewritten as the sum of the terms which contribute towards enstrophy production (i.e. − (*PQ*_w_ − *ω_i_S_ij_ω_j_*/4)) and dissipation rate generation (i.e. *S_ij_S_jk_S_ki_*/3) as follows:

3.1 $$\begin{array}{rl} R & =\frac{1}{3}(-P^{3}+3PQ-S_{ij}S_{jk}S_{ki})-\frac{1}{4}\omega_{i}S_{ij}\omega_{j}\\ & =\underset{R_{s}}{\underbrace{\frac{1}{3}(-P^{3}+3PQ_{S}-S_{ij}S_{jk}S_{ki})}}+PQ_{W}-\frac{1}{4}\omega_{i}S_{ij}\omega_{j}. \end{array}$$

Away from the flame front, where *P* ≈ 0, it is possible to approximate *R*_S_ ≈ −*S_ij_S_jk_S_ki_*/3 > 0 and *PQ*_W_ − *ω_i_S_ij_ω_j_*/4 ≈ −*ω_i_S_ij_ω_j_*/4 < 0. Hence, where *P* ≈ 0, R* will be non-zero where there is an imbalance of − *ω_i_S_ij_ω_j_*/4 and − *S_ij_S_jk_S_ki_*/3. It is evident from figure 2 that, in case A, this imbalance is present across the entire domain, on both sides of the flame front, whereas in cases B–E it is significant only in the unburned gas region and is negligible elsewhere.

**Figure 3.**
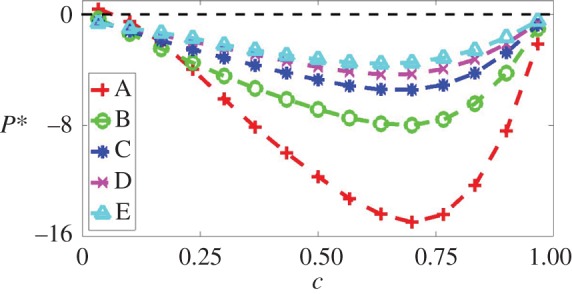
Variations of the mean values of $P^{*}=P\times \delta_{\mathrm{th}}/S_{L}$ conditional upon *c* for cases A (red +), B (green o), C (blue *), D (magenta x) and E (cyan triangle).

**Figure 4.**
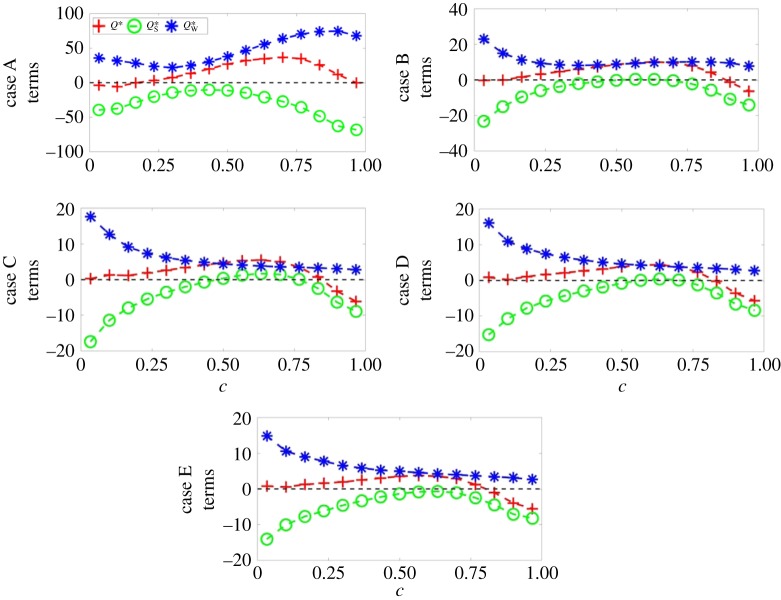
Variations of the mean values of $Q^{*}=Q\times {\left(\delta_{\mathrm{th}}/S_{L}\right)}^{2}$ (red +), $Q_{S}^{*}=Q_{S}\times {\left(\delta_{\mathrm{th}}/S_{L}\right)}^{2}$ (green o) and $Q_{W}^{*}=Q_{W}\times {\left(\delta_{\mathrm{th}}/S_{L}\right)}^{2}$ (blue *) conditional upon *c* for (top to bottom) cases A–E.

**Figure 5.**
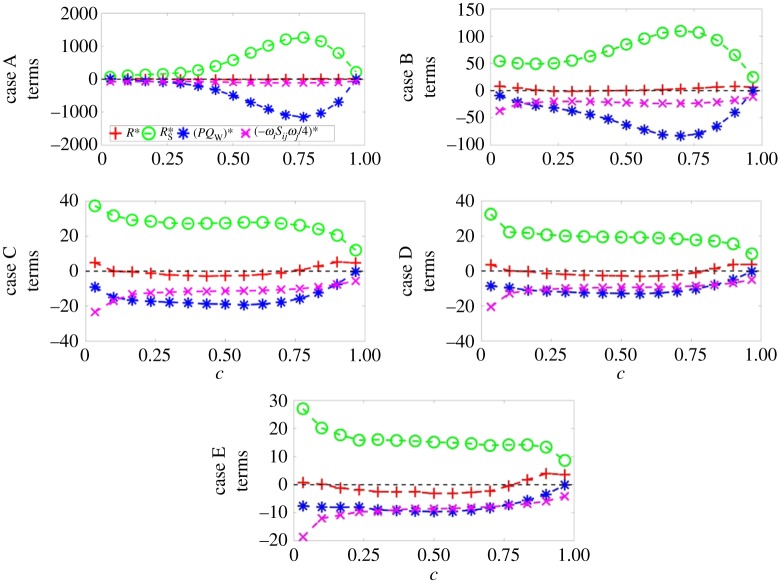
Variations of the mean values of $R^{*}=R\times {\left(\delta_{\mathrm{th}}/S_{L}\right)}^{3}$ (red +), $R_{S}^{*}=R_{S}\times {\left(\delta_{\mathrm{th}}/S_{L}\right)}^{3}$ (green o), (*PQ*_W_)* = *PQ*_W_ × (*δ*_th_/*S*_L_)^3^ (blue *) and ${\left(-\omega_{i}S_{ij}\omega_{j}/4\right)}^{*}=(-\omega_{i}S_{ij}\omega_{j}/4)\times {\left(\delta_{\mathrm{th}}/S_{L}\right)}^{3}$ (magenta x) conditional upon *c* for (top to bottom) cases A–E.

The variations of the normalized mean values of the three invariants (*P*, *Q*, *R*) and of their constituent terms conditional on *c* are shown in figures 3–5. In general, in turbulent premixed flames ∇⋅u=−P remains positive. However, locations of negative dilatation rate can develop. Such locations are most likely to develop where the flame is convex to the reactants (figure 2). In all cases considered here, the probability of finding positive dilatation rates dominates and, thus, the mean values of *P* remain negative across most of the flame front (figure 3). In the *Le* < 1.0 flames, the reactants diffuse faster into the reaction zone than the rate of thermal diffusion out of it, which leads to the simultaneous occurrence of high temperature and reactant concentrations, giving rise to an increase in the burning rate and in the magnitude of the dilatation rate |∇⋅u|=|−P|. The reaction progress variable *c*-isosurfaces on the unburned gas side are so strongly wrinkled in case A that it shows a considerable probability of finding zones that are so strongly convexly curved towards the reactants that the dilatation rate ahead of these zones assumes large negative values because of the intense defocusing of heat. This gives rise, in case A, to a weakly positive mean value of *P* conditional on *c* towards the unburned gas side of the flame.

The similarities between the distributions of Q* apparent from figure 2, column 3, in cases B–E in contrast to that of case A are mirrored by the mean variation of *Q* and its constituent terms with *c*, as can be seen in figure 4. The mean variations of *Q*, *Q*_S_ and *Q*_W_ conditional on *c* in case A differ from those in cases B–E in several noticeable ways. However, these differences are not apparent for all values of *c*. For values of *c* < 0.35, all cases show somewhat similar behaviour: for *c* close to zero, the mean values of *Q*_S_ (negative) and *Q*_W_ (positive) approximately balance each other such that the mean value of *Q* assumes a negligible value and, as *c* increases, the mean values of *Q*_S_ and *Q*_W_ both tend towards 0. However, the magnitude of the mean value of *Q*_S_ decreases more rapidly than that of *Q*_W_, such that over this subrange (*c* < 0.35) *Q* rises monotonically with *c*. The dissipation rate of kinetic energy *ε* = (*τ_ij_*∂*u_i_*/∂*x_j_*)/*ρ* can be expressed as *ϵ* = *ν*(4*P*^2^/3 − 4*Q*_S_), which indicates that *Q*_S _= 0.25(4*P*^2^/3 − ε/*ν*) assumes negative (positive) values in the dissipation (dilatation)-dominated regions. Following Wacks & Chakraborty [23], *Q*_S_ may be subdivided as $Q_{S}=Q_{S1}+Q_{S2}=P^{2}\text{/}3-\epsilon \text{/}4\nu$. The density for the unity Lewis number low Mach number flames can be expressed as $\rho =\rho_{0}\text{/( }1+\tau c\text{) }$ (where *ρ*_0_ is the unburned gas density), which upon combining with the mass conservation equation yields $\nabla \cdot u=\tau \rho S_{d}|\nabla c|\text{/}\rho_{0}$ [55,56]. It has been shown elsewhere [56] that, although $\nabla \cdot u=\tau \rho S_{d}|\nabla c|/\rho_{0}$ does not remain strictly valid for Le≠1, ∇⋅u still scales with $\tau \rho S_{d}|\nabla c|/\rho_{0}$ (i.e. $\nabla \cdot u∼\tau \rho S_{d}|\nabla c|\text{/}\rho_{0}$). The quantities *ρS*_d_ and |∇_c_| can be scaled with *ρ*_0_*S*_L_ and (1/*δ*_th_), respectively, which lead to $\nabla \cdot u∼\tau S_{L}\text{/}\delta_{\mathrm{th}}$. Thus, it is possible to write $Q_{S1}=P^{2}/3∼{\left\{\tau S_{L}/\delta_{\mathrm{th}}\right\}}^{2}\;$ and $|Q_{S2}|=|-\epsilon /4\nu |∼1/\tau_{\eta }^{2}$ (where *τ_η_* is the Kolmogorov time scale), which leads to $Q_{S1}/|Q_{S2}|∼\tau^{2}Ka^{-2}$. This suggests that the magnitude of *Q*_S1_ in comparison with *Q*_S2_ is expected to be small for large values of *Ka* (*Ka* ≫ 1), and thus the mean behaviour of *Q*_S_ is principally governed by *Q*_S2_ = −ϵ/4*ν* for the cases considered here. The contribution of *Q*_S_ is principally governed by *S_ij_S_ij_* in the unburned gas region, where the magnitude of the dilatation rate is small. However, *P*^2^ assumes high values because of large values of the dilatation rate as the reaction zone is approached (figure 3), and thus the contribution of *P*^2^ compensates *S_ij_S_ij_* to give rise to a small magnitude of the mean value of *Q*_S_ for intermediate values of *c*. The effects of *P*^2^ weaken on the burned gas side and thus the mean behaviour of (−*S_ij_S_ij_*)/2 determines the mean behaviour of *Q*_S_, and yields predominantly negative values on the burned gas side of the flame brush. In case A, *Q*_W_ = *W_ij_W_ij_*/2 = *ω_i_ω_i_*/4 begins to increase in magnitude after *c *= 0.35 and continues to increase monotonically with *c* until *c* ≈ 0.95. This behaviour arises as a result of flame-generated vorticity due to strong baroclinic torque induced by strong flame normal acceleration in case A. Interested readers are referred to Chakraborty *et al.* [12] for further discussion. The flame-generated vorticity generation weakens with increasing *Le* [12], and in cases C–E the mean value of *Q*_W_ = (*W_ij_W_ij_*)/2 decreases monotonically from the unburned to the burned gas side. The flame-generated vorticity in case B is not as strong as that in case A and therefore it does not exhibit an increase in the mean value of $Q_{W}=(W_{ij}W_{ij})\text{/}2$, but it is sufficient to maintain an approximately constant mean positive value of *Q*_W_ for *c *> 0.35.

As a result of the aforementioned behaviour of *Q*_W_, the maximum mean value attained by *Q* is higher for case A than for any other case and is located at a higher value of *c* than in any other case. This feature could already be anticipated from figure 2 by considering the size, magnitude and location of the highly positive regions visible in case A: these regions are larger in size, higher in magnitude and extend further into the burned gas region than similar regions in cases B–E. It should be pointed out that the magnitude of the mean value of *Q* diminishes from case A to the other cases, which accentuates the difference in the magnitude of the terms. Finally, the increase in the magnitude of *Q*_W_ in case A results in the increase in the mean value of *Q* remaining vorticity dominated across almost the entire flame (except for a very small region near *c* = 0), whereas cases B–E, which do not benefit from flame-generated vorticity to the same extent as case A, all possess regions with mean *Q* < 0. These regions with negative mean *Q* are located away from the unburned gas side (i.e. in all cases the vorticity dominates towards the unburned gas side); however, the value of *c* which signifies the onset of these regions decreases with increasing *Le*.

Figure 5 shows the mean variation of normalized *R* and its components (*R*_S_, *PQ*_W_ and $-\omega_{i}S_{ij}\omega_{j}/4$) conditional upon *c* for cases A–E. In all cases the mean value of *R* remains close to zero across the entire flame, indicating that the terms are well balanced across the entire flame. More specifically, in all cases *R*_S_ remains positive and is balanced by the negative contributions which arise due to both *PQ*_W_ and $-\omega_{i}S_{ij}\omega_{j}/4$ (this follows from the fact that *P* and *Q*_W_ are predominantly negative and positive, respectively, within the flame front and the vortex-stretching term *ω_i_S_ij_ω_j_* in the mean sense generates enstrophy (i.e. remains positive)). However, the relative importance of the contributions arising due to *PQ*_W_ and − *ω_i_S_ij_ω_j_*/4 changes with increasing Lewis number, such that for low Lewis number flames (e.g. *Le *= 0.34 ) the magnitudes of the mean contributions due to *R*_S_ and *PQ*_W_ remain much greater than the magnitude of the mean values of − *ω_i_S_ij_ω_j_*/4, whereas for high Lewis number flames (e.g. *Le *≈ 1.0 ) these contributions are of approximately equal importance. Furthermore, the size of the dominating constituent terms decreases by one order of magnitude from case A to case B and by a further order of magnitude from case B to cases C–E. The greater (negative) magnitudes of *R*_S_ and *PQ*_W_ observed in cases A and B arise from the augmented dilatation rate experienced by these flames, as can be seen in figure 3, especially near *c* ≈ 0.75.

**Figure 6.**
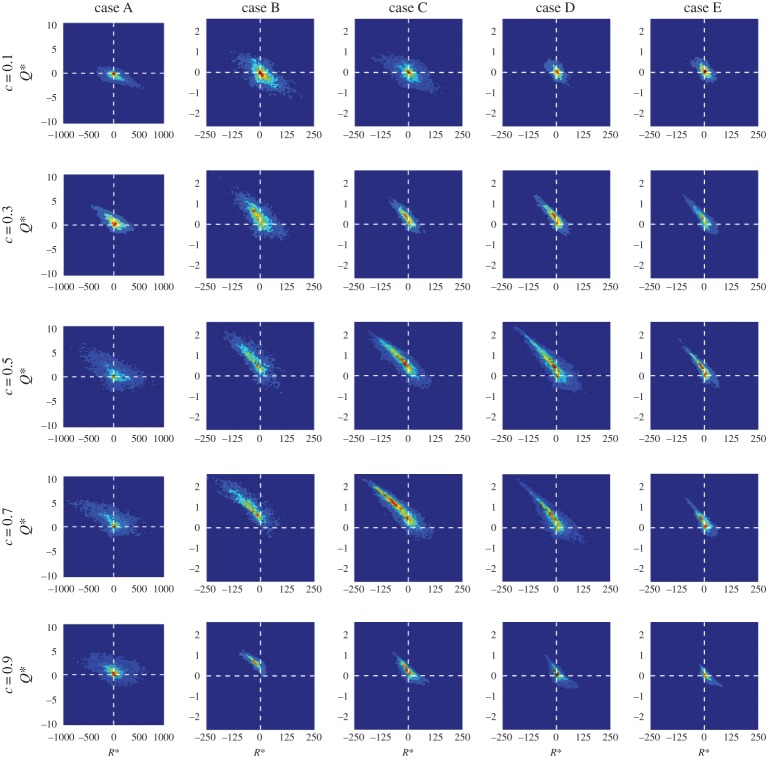
Joint PDFs of $Q^{*}=Q\times {\left(\delta_{\mathrm{th}}/S_{L}\right)}^{2}$ and R* = *R* × (*δ*_th_/*S*_L_)^3^, $\text{PDF}(Q^{*},R^{*})$, on *c*-isosurfaces (top to bottom) *c* = 0.1, 0.3, 0.5, 0.7, 0.9 for (left to right) cases A–E. The value of $\text{PDF}(Q^{*},R^{*})$ rises from blue to red colour.

Figure 6 shows the joint PDF of the normalized second and third invariants, PDF(Q*, R*), for cases A–E on the isosurfaces *c *= 0.1, 0.3, 0.5, 0.7, 0.9 in order to illustrate the nature of the distribution of samples in the *Q*−*R* plane and to analyse the correlation between Q* and R*.^1^ The joint PDF exhibits a negative correlation between Q* and R* [26,27] in all cases and on all *c*-isosurfaces which are considered in this analysis: R* increases as Q* decreases. However, while, in general, all *c*-isosurfaces and all cases exhibit negative correlations, the strength of the negative correlation is noticeably less in case A than in cases B–E (i.e. the downwards slope of the distribution is greater for cases B–E than for case A). Note that the ratio of the ranges of the abscissa and ordinate are the same for all cases and thus the slopes may be compared. In addition, the distribution of the data is wider (i.e. the data are more spread out) in case A than in cases B–E. It is also worth noting that, although Q* and R* are negatively correlated, most non-negligible values lie in the top left quadrant of the joint PDF or close to the origin (i.e. Q* = 0 and R* = 0). In other words, although a decrease in Q* is associated with a shift from the combined dilatation- and vorticity-dominated regions to strain-dominated regions and an increase in R* is associated with a shift towards increased dominance of *R*_S_ over (*PQ*_W_ − *ω_i_S_ij_ω_j_*/4), nevertheless the joint PDF remains negligible throughout most of the Q* < 0 and R* > 0 quadrant. According to the previous analyses [21,25,36] the observed features of the joint PDF suggest that the S2 and S4 topologies are expected to play significant roles in all cases considered here.

**Figure 7.**
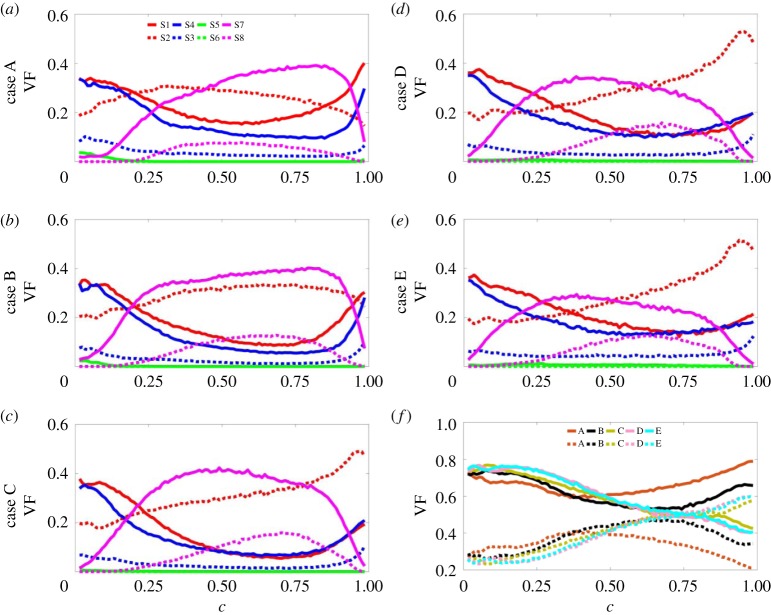
(*a*–*e*) Variation in the VFs of topologies S1–S8 with reaction progress variable *c* for cases A–E: focal topologies S1, S4, S5, S7 (red, blue, green, magenta solid lines, respectively) and nodal topologies S2, S3, S6, S8 (red, blue, green, magenta dotted lines, respectively). (*f*) Variation in the VFs of the total focal (solid lines) and nodal (dotted lines) topologies with *c* for cases A–E (brown, black, olive, pink, cyan, respectively).

The variation of the population of each individual local flow topology, S1–S8, across the flame and how these variations vary from case to case is considered in figure 7. Following the approach adopted by Cifuentes *et al.* [41,42], figure 7*a*–*e* shows the behaviour of the volume fraction (VF) of each topology as a function of *c*. It can be seen from figure 7 that the behaviour of the distributions of the local flow topologies is very much dependent on the Lewis number. Topologies S1–S4 are present for all values of *P* (figure 1) and, consequently, exhibit non-negligible values across the entire flame brush. However, each topology responds differently to the effect of the changing Lewis number. In case A, topologies S1 and S4 exhibit local maxima at both low and high value of *c* (i.e. *c* ≈ 0.0 and *c* ≈ 1.0) with the VF of S1 assuming higher values than that of S4 for all values of *c*. As *Le* increases (i.e. from case B to case E) little effect can be seen at lower values of *c*, but at higher values of *c* the profiles of S1 and S4 collapse onto one curve and the local maximum at *c* ≈ 1.0 decreases in value in both cases. By contrast, topology S2 for case A exhibits local minima at both low and high values of *c* and as *Le* increases the local minimum at *c* ≈ 1.0 increases in value, developing into the maximum. Finally, topology S3, which assumes the smallest values of VF of S1–S4 across the flame brush, appears to be largely unaffected by the changing value of the Lewis number. Topologies S5 and S6 are associated with negative values of the dilatation rate (∇⋅u=−P<0, figure 1) and are largely absent in combustion (figure 3). Hence, the likelihood of finding either of these topologies remains negligible (but non-zero) across the entire flame brush for all values of Lewis number. Conversely, topologies S7 and S8 are associated with positive values of the dilatation rate (∇⋅u=−P>0, figure 1) and, as has been seen clearly in figure 3, they assume non-negligible values for intermediate values of *c*. The magnitude and profile of the VFs for topology S7 show little variation with Lewis number, although the location of the maximum value shifts from high *c* for low Lewis number (case A, *c *≈ 0.85 ) to low *c* for high Lewis number (case E, *c* ≈ 0.35). In other words, the likelihood of the occurrence of S7 remains approximately constant despite changes in the value of *Le*, notwithstanding the noticeable increase in the magnitude of the dilatation rate with increasing *Le* observed in figure 3. Finally, in all cases the profile of topology S8 exhibits a maximum value at some intermediate *c*-value. In case A, the profile of the VF is somewhat flatter and of lesser magnitude than in the other cases.

Figure 7*f* shows the variation with *c* for all cases A–E of the VFs of the total combined focal (i.e. S1, S4, S5, S7) and nodal (i.e. S2, S3, S6, S8) topologies. In all cases, focal topologies are dominant in the unburned gas region (for *c *< 0.7). Within this region, for 0.1 < *c* < 0.5, the VFs of focal topologies increase with increasing Lewis number with cases D and E equally dominant. Thereafter, for *c* > 0.5, the order reverses such that the VFs of focal topologies decrease with increasing Lewis number. This is due to flame-generated turbulence, which is much greater in low Lewis number cases and leads to an increase in the number of vortical structures (related to focal topologies) present in the flow. Consequently, for *c* > 0.7, in cases C–E the VFs for the focal topologies have fallen to such an extent that the VFs for the nodal topologies become dominant, while in cases A and B the VFs for the focal topologies continue to rise. The results obtained here for cases C–E, where the VFs of focal topologies decrease from the unburned to the burned gas side, are in agreement with previous simple chemistry analyses [41,42], whereas the observed behaviour of cases A and B differs because of the effects of flame-generated vorticity due to the strong baroclinic torque [12] in low Lewis number combustion.

**Figure 8.**
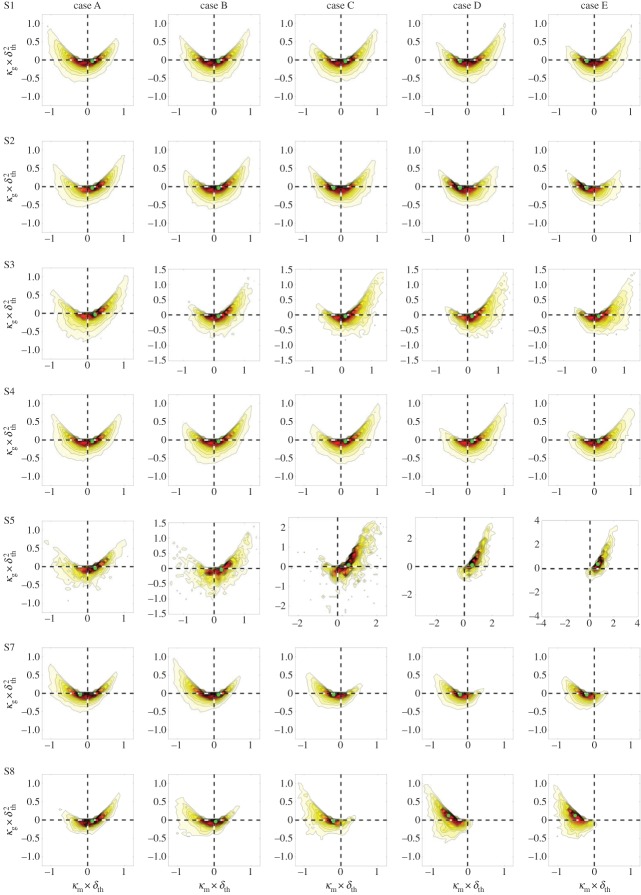
Joint PDF of the normalized mean and Gaussian curvatures coloured by count for (left to right) cases A–E. Data for S6 are not shown because of insufficient data. The magnitude increases with colour from white to red. Green dots indicate the location of the maximum values.

The statistical dependence of the flame curvature on the local flow topologies is examined next. Following Dopazo *et al.* [31], the curvature of each *c*-isosurface is assessed with respect to its mean ($\kappa_{m}=(\kappa_{1}+\kappa_{2})/2=1/2\nabla \cdot (-\nabla \text{c}/|\nabla \text{c}|)$) and Gauss (*κ*_g_ = *κ*_1_*κ*_2_) curvatures, where the principal curvatures are denoted *κ*_1_ and *κ*_2_ [31,42]. Complex, non-physical curvatures, such as the region $\kappa_{g}>\kappa_{m}^{2}$, are discounted. Those wrinkles which are convex to the reactants are said to exhibit positive mean curvature, while those which are concave to the reactants are said to exhibit negative mean curvature (figure 2). Then {*κ*_m_ > 0, *κ*_g_ > 0} represents cup-convex and $\left\{\kappa_{m}<0,\kappa_{g}>0\right\}$ cup-concave flame topology; {*κ*_m_ > 0, *κ*_g_ < 0} represents saddle-convex and $\left\{\kappa_{m}<0,\kappa_{g}<0\right\}$ saddle-concave flame topology; and {*κ*_m_ > 0, *κ*_g_ = 0} represents tile-convex and $\left\{\kappa_{m}<0,\kappa_{g}=0\right\}$ tile-concave flame topology. Figure 8 shows the joint PDFs between *κ*_m_ and *κ*_g_ for cases A–E conditional on each local flow topology. Topology S6, associated with positive values of the dilatation rate, is not shown because of the lack of available data points for this topology. An examination of figure 1*a*(i) reveals that the region corresponding to S5 is much larger than that corresponding to S6. Thus, S5 is relatively more prevalent than S6. For this reason, there are sufficient data points to demonstrate the behaviour of S5 in the *κ*_m_ − *κ*_g_ plane, but not that of S6. The plots in figure 8 are coloured to highlight the highest concentrations of data points. The actual values of the joint PDF to which the colours correspond is of no interest because the population of different topologies varies widely and the actual values would be of little use. It is the shape of the distribution and its relative spread which hold useful information. It is evident from figure 8 that the distributions associated with the different topologies and Lewis numbers exhibit appreciably different behaviour. In general, case A exhibits the most symmetric distributions for all topologies in comparison with the other cases. The symmetry of the distribution tends to break down with increasing Lewis number. Topology S5, associated with negative dilatation rates, exhibits strong cup-convex curvature $\text{( }\kappa_{m}>0,\kappa_{g}>0)$ at higher Lewis number (cases C–E), whereas topologies S7 and S8, which are associated with positive dilatation rates, exhibit strong cup-concave curvature (*κ*_m_ < 0, *κ*_g_ < 0) for the same cases. This behaviour originates from the negative correlation between ∇⋅u and *κ*_m_ in the cases with *Le* ≈ 1.0 (e.g. cases C–E) [57] due to focusing (defocusing) of heat at negative (positive) curvature locations, which leads to high positive values of ∇⋅u at negative values of *κ*_m_, whereas small positive and negative values of ∇⋅u are obtained for positive values of *κ*_m_. The above effect is to some extent countered in the *Le* < 1 flames because high temperature values are associated with the flame wrinkles with *κ*_m_ > 0 [13,14,18], which also tends to increase the local value of ∇⋅u. Thus, topologies S7 and S8, which are associated with ∇⋅u=−P>0, are more prevalent for *κ*_m_ < 0 for cases C–E with *Le *≈ 1.0, but the probability of occurrence of S7 and S8 becomes more symmetric with respect to *κ*_m_ for case A with *Le* = 0.34. The topology S5, which is obtained only for ∇⋅u=−P<0, occurs predominantly for *κ*_m_ > 0 for all cases. Topologies S1–S4 exhibit more symmetric distributions, owing to the contributions from all values of *P*, although S3 and S4 are somewhat skewed towards cup-convex for high *Le* cases (cases C–E) because S3 and S4 can occur also for ∇⋅u=−P>0 at the positive value of *κ*_m_.

Figure 1 shows the generic flow structures which are associated with each of the local flow topologies. Turbulent processes such as micro-mixing (characterized by the SDR, $N_{c}=D\nabla c\cdot \nabla c$) and enstrophy (Ω=ω⋅ω/2) transport are dependent on the local flow structure and, hence, on the local flow topology. Thus local flow topologies are of fundamental importance in understanding these processes. Following Chakraborty [58], Chakraborty *et al.* [59] and Tsinober *et al.* [60], the transport equations for *N*_c_ and Ω may be written as

3.2*a* $$\begin{array}{rl} \rho \frac{\text{D}N_{c}}{\text{D}t} & =\frac{\partial }{\partial x_{j}}\left(\rho D\frac{\partial N_{c}}{\partial x_{j}}\right)-2D\frac{\text{D}c}{\text{D}t}\frac{\partial c}{\partial x_{k}}\frac{\partial \rho }{\partial x_{k}}-2\rho D\underset{\Lambda }{\underbrace{\frac{\partial c}{\partial x_{i}}\frac{\partial u_{i}}{\partial x_{j}}\frac{\partial c}{\partial x_{j}}}}\\ & \;+2D\frac{\partial {\dot{\omega }}_{T}}{\partial x_{k}}\frac{\partial c}{\partial x_{k}}-2\rho D^{2}\frac{\partial^{2}c}{\partial x_{k}\partial x_{i}}\frac{\partial^{2}c}{\partial x_{k}\partial x_{i}}+f(D), \end{array}$$

3.2*b* $$\begin{array}{rl} \frac{\text{D}\Omega }{\text{D}t} & =\underset{V}{\underbrace{\omega_{i}\omega_{k}\frac{\partial u_{i}}{\partial x_{k}}}}-\epsilon_{ijk}\omega_{i}\frac{1}{\rho^{2}}\frac{\partial \rho }{\partial x_{j}}\frac{\partial \tau_{kl}}{\partial x_{l}}\\ & \;+\frac{\epsilon_{ijk}\omega_{i}}{\rho }\frac{\partial^{2}\tau_{kl}}{\partial x_{j}\partial x_{l}}-2\frac{\partial u_{k}}{\partial x_{k}}\Omega +\epsilon_{ijk}\frac{\omega_{i}}{\rho^{2}}\frac{\partial \rho }{\partial x_{j}}\frac{\partial p}{\partial x_{k}}. \end{array}$$

In these equations *f*(*D*) represents the contribution due to diffusivity gradients. $\tau_{ij},{\dot{\omega }}_{T}$ and *p* are the viscous stress tensor, chemical source term and pressure, respectively. −2*ρDΛ* is the scalar--turbulence interaction term and *V* is the vortex-stretching term [15,16,43,44,58–60]. The angles described by ∇c and ω and the eigenvectors associated with $e_{\alpha }$, $e_{\beta }$ and $e_{\gamma }$, where *e_α_*, $e_{\beta }$ and *e_γ_* are the most extensive (positive), intermediate and the most compressive (negative) strain rates, respectively, are written as {α,β,γ} and {*α*′, *β*′, *γ*′}, respectively. The scalar--turbulence interaction term, $\Lambda =(\partial c/\partial x_{i})(\partial u_{i}/\partial x_{j})(\partial c/\partial x_{j})$, may then be written in terms of the angles {*α*, *β*, *γ*}: $\Lambda =(e_{\alpha }\cos^{2}\alpha +e_{\beta }\cos^{2}\beta +e_{\gamma }\cos^{2}\gamma )\nabla c\cdot \nabla c=a_{n}\;\nabla c\cdot \nabla c$, where $a_{n}=N_{i}N_{j}\partial u_{i}/\partial x_{j}\;$ is the normal strain rate with $N_{i}=-(\partial c/\partial x_{i})/|\nabla c|$ being the *i*th component of the flame normal vector [15,16,43,44]. In other words, the behaviours of *Λ* and *a_n_* are governed by the alignment of ∇c with the local principal strain rates and the sign of *Λ* depends on that of *a_n_* [15,16,43,44]. The vortex-stretching term, *V* = *ω_i_ω_k_*∂*u_i_*/∂*x_k_*, may similarly be expressed in terms of the angles $\left\{\alpha^{\prime },\beta^{\prime },\gamma^{\prime }\right\}$:*V* = 2(*e_α_* cos^2^*α*′ + e*_β_* cos^2^*β*′ + e*_γ_* cos^2^*γ*′)Ω.

In the light of these expressions for *Λ* and *V*, it is clear that the statistical behaviours of the scalar--turbulence interaction and the vortex-stretching terms should exhibit dependence on the local flow topologies, which represent particular combinations of strain rate and vorticity distributions.

**Figure 9.**
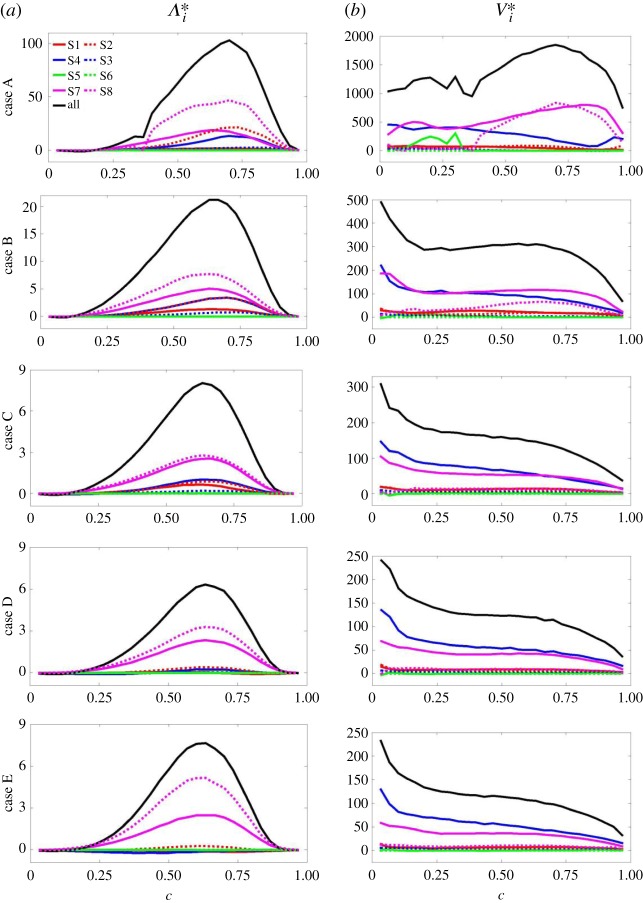
Variation with reaction progress variable *c* of the mean values of (*a*) $\Lambda_{i}^{*}=\Lambda_{i}\times \;\delta_{\mathrm{th}}^{3}/S_{L}$ and (*b*) $V_{i}^{*}=V_{i}\times {\left(\delta_{\mathrm{th}}/S_{L}\right)}^{3}\;$for (top to bottom) cases A–E, respectively, where {i=0} is the total value of the terms (black lines) and {*i* = 1, … , 8} are the percentage-topology-weighted terms corresponding to S1–S8: focal topologies S1, S4, S5, S7 (red, blue, green, magenta solid lines, respectively) and nodal topologies S2, S3, S6, S8 (red, blue, green, magenta dotted lines, respectively).

Figure 9 shows the contribution of different topologies towards the mean values of *Λ* and *V* conditional on *c*, where $\Lambda =\Lambda_{0}=\sum_{i=1}^{8}\Lambda_{i}$ and $V=V_{0}=\sum_{i=1}^{8}V_{i}$ are the total scalar--turbulence interaction and vortex-stretching terms, respectively, and *Λ_i_* and *V_i_* are the contributions which arise due to each individual topology. It is evident (figure 9*a*) that in all cases the scalar--turbulence interaction term remains positive across most of the flame brush and attains its maximum value at *c* ≈ 0.7. It is, furthermore, evident from figure 9 that the lead contributor towards the behaviour of $\Lambda =\Lambda_{0}$ is *Λ*_8_ (i.e. the contribution arising from topology S8). The magnitude of the scalar--turbulence interaction term *Λ* originating from topology S7 varies between 50% and 100% of that of S8 and is the second largest contributor. Topologies S7 and S8 are associated with regions of high dilatation rate and the overall behaviour of *Λ* is clearly dependent on its behaviour in these regions. This is further emphasized by the magnitudes of the peak value of *Λ*, which are greatest for cases A and B where the high burning rates are obtained due to the low Lewis number in these cases. Even topologies S1–S4 (which are present for all values of *P* and not only where the dilatation rate is positive) exhibit non-negligible contributions to the scalar--turbulence interaction term for values of *c*, which typically lie within the reaction zone (i.e. medium values of *c*) and in cases where the value of the Lewis number is relatively low (i.e. cases A–C). It has been shown elsewhere [15,16,43,44,61,62] that a preferential alignment between ∇c and $e_{\alpha }$ (*e_γ_*), which is characterized by the high probability of $\cos^{2}\alpha \approx 1.0$ ($\cos^{2}\gamma \approx 1.0$), leads to positive (negative) *Λ*. It is worth noting that ∇c preferentially aligns with *e_α_* ($e_{\gamma }$) when the strain rate induced by flame normal acceleration overcomes (is overcome by) turbulence straining [15,16,43,44,61,62]. The uniformly positive values assumed by *Λ*_0_ in all cases show that the heat release is sufficiently strong even in the highest Lewis number case considered here (Le=1.2) to ensure alignment between ∇c and *e_α_*. However, the increased heat release enjoyed by the lower Lewis number cases engenders an increase in the magnitude of $\Lambda_{0}$ and those topologies which are associated with high dilatation rates (e.g. S7 and S8), as noted above.

Figure 9*b* shows the contribution of different topologies (*V_i_*) to the mean values of the vortex-stretching term *V* = *V*_0_ conditional on *c*. This figure reveals that the mean value of $V=V_{0}$ remains positive in all cases A–E. This feature is evident also for individual topologies, which remain largely positive across the entire flame brush. The predominant alignment of ***ω*** with the intermediate and most extensive principal strain rates ($e_{\beta }$ and *e_α_*) in these cases, in accordance with previous findings [58,60], gives rise to the positive mean value of *V* for all cases considered here, although the magnitude of the value of *V* increases with decreasing Lewis number because of the increased flame-generated turbulence experienced by the low Lewis number flames.^2^ In case A, this phenomenon leads to the development of a maximum at c≈0.7 due to flame-generated enstrophy, as opposed to the decay of enstrophy evident in the higher Lewis number cases (cases C–E) as the flame is traversed (i.e. as *c* increases from 0 to 1). Case B exhibits intermediate behaviour, in which the decay of enstrophy is arrested, but flame-generated enstrophy is insufficient to lead to any noticeable increase in *V* = *V*_0_. In case E, the leading contributor towards *V* = *V*_0_ is the focal topology S4 and the secondary contributor is another focal topology S7. No other topologies make significant contributions towards *V* = *V*_0_. As the Lewis number decreases (from case D to case A), these positions are reversed such that S7 becomes the primary contributor (due to the increased flame-generated enstrophy in low Lewis number flames) and S8 also becomes more prominent (cases A and B) until its magnitude matches that of S7 (case A). The predominance of S4 and S7 for all values of Lewis number considered here is due to the focal nature of these topologies, which is associated with vortex stretching (figure 1). This natural proclivity for vortex stretching dominates in the absence of significant flame-induced enstrophy generation and is subject to decay as *Ω* decreases within the flame, as has been noted in cases C–E, such that the peak mean values of $V_{0},\;\;V_{4}$ and *V*_7_ are all obtained close to c≈0.0. In cases A and B, the behaviour of $V_{4}$ remains unaltered, but that of *V*_7_ is affected by the significant flame-induced turbulence in the regions where heat release is strong. The behaviour of $V_{8}$ arising from nodal topology S8 is due to the same effect, such that *V*_8_ remains negligible where heat release is negligible, but shows the same features as $V_{7}$ in the regions where heat release is strong. The overall behaviour of the vortex-stretching term *V*_0_ is thus attributable to two factors: (i) strong contributions due to focal topologies and (ii) areas of strong heat release regardless of the focal or nodal nature of the topology.

## 4. Conclusion

Three-dimensional DNSs of freely propagating statistically planar premixed turbulent flames have been analysed to investigate the effects of Lewis number on the behaviour of the three invariants of the velocity gradient tensor (P,Q,R) and the consequent eight individual local flow topologies (S1–S8). The range of Lewis numbers considered herein (Le=0.34,0.6,0.8, 1.0 and 1.2) includes cases where mass diffusivity dominates (Le<1.0) and where thermal diffusivity dominates (*Le* > 1.0). The lowest Lewis number case (case A) exhibited strong signs of increased burning rates and flame-generated enstrophy. These were embodied by a larger magnitude of the dilatation rate (related to the first invariant), of the $Q_{W}$-component associated with vorticity-dominated regions (related to the second invariant) and of the $PQ_{W}$-component associated with both the dilatation rate and vorticity (related to the third invariant). This was also apparent from the variation of the individual flow topologies across the flame, such that those flow topologies associated with a positive dilatation rate (S7 and S8) were more prominent further into the flame (i.e. at higher values of *c*) for low Lewis number cases (i.e. cases A and B). Although heat release due to combustion was strong enough in all cases to ensure the preferential alignment of the scalar gradient (∇c) with the most extensive strain rate (*e_α_*), those cases (i.e. cases A and B) and topologies (S7 and S8) which experienced augmented heat release exhibited greatly increased peak magnitudes of the scalar--turbulence interaction term in the region of the flame associated with a non-negligible dilatation rate. Likewise, the behaviour of the vortex-stretching term in the burned gas region for the low Lewis number cases (i.e. cases A and B) was shown to depend significantly on contributions arising from the topologies associated with a positive dilatation rate (S7 and S8) and areas of strong heat release (c≈0.7). The joint PDF of the second and third invariants showed a similar general behaviour for all Lewis numbers, although minor differences were noted. The interaction between the flow and flame topologies has been analysed in terms of the joint PDFs of the mean and Gaussian curvatures (i.e. *κ*_m_ and *κ*_g_) conditional on flow topologies. These joint PDFs exhibited greater symmetry along the $\kappa_{m}=0$ axis for lower Lewis number flames. The focusing (defocusing) of heat at *κ*_m_ < 0 (*κ*_m_ > 0) leads to a high positive (low positive or negative) dilatation rate in the *Le* ≈ 1.0 flames, which leads to the preferential occurrence of flow topologies specific to a positive (negative) dilatation rate at the flame locations with *κ*_m_ < 0 (*κ*_m_ > 0). This directionality weakens for flames with Le<1.0 because this tendency is countered by the presence of high temperature zones at the positively curved zones, which locally give rise to a high positive dilatation rate at *κ*_m_ > 0. Thus, the probability of the occurrence of flow topologies for positive and negative mean curvatures is more symmetric for Le<1.0 flames than for the corresponding distributions for the *Le* ≈ 1.0 flames. This suggests that the dominant flow topologies for a curved flame might be different from a planar flame and the global Lewis number is likely to have a significant influence on this interaction of flow and flame topologies in premixed turbulent premixed flames.

Figure 1 shows that each of these eight flow topologies is associated with a canonical flow configuration. Thus, the distributions of the topologies and their contributions to scalar--turbulence interaction and vortex-stretching terms in the SDR and enstrophy transport equations, respectively, could, in principle, be used to design simplified experimental and computational configurations based on dominant flow topology contributions for different characteristic Lewis numbers. This will offer guidance for choosing representative simple flow geometries for the development of turbulence and combustion models (because the SDR closure can be linked to the mean/filtered reaction rate modelling and the mean enstrophy transport can be linked to the dissipation rate of the kinetic energy closure) and their validation based on experiments and also using RANS simulations and LES for turbulent premixed combustion for different values of *Le*. The high-fidelity turbulence and combustion models identified based on the aforementioned exercise are expected to give rise to the development of methodologies for accurate quantitative predictions of burning rate and pollutant emission in future combustion devices in the presence of differential diffusions of heat and mass.

## Supplementary Material

Matlab files

## Supplementary Material

Figure files

## Footnotes

1 The actual values of the joint PDF are not important for the subfigures in figure 6 because the purpose of these plots is to illustrate the relative population of samples in *Q *− *R* space and the correlation between *Q* and *R*.

2 The physical reasons for increased flame-generated turbulence for low Lewis number flames have been discussed elsewhere [12] and are not repeated here. Moreover, Lewis number dependence on the alignment of ***ω*** with the intermediate and most extensive principal strain rates has been explained in [58].

## Data accessibility

The datasets and code supporting this article have been uploaded as the electronic supplementary material.

## Appendix A. Non-dimensional form of conservation equations

The non-dimensional mass, momentum, energy and progress variable transport equations are given as

A 1 $$\begin{array}{rl} \frac{\partial \rho^{+}}{\partial t^{+}}+\frac{\partial (\rho^{+}u_{i}^{+})}{\partial x_{i}^{+}} & =0, \end{array}$$

A 2 $$\begin{array}{rl} \frac{\partial (\rho^{+}u_{i}^{+})}{\partial t^{+}}+\frac{\partial (\rho^{+}u_{k}^{+}u_{i}^{+})}{\partial x_{k}^{+}} & =-\frac{\partial p^{+}}{\partial x_{i}^{+}}+\frac{1}{\text{Re}}\frac{\partial (\tau_{ki}^{+})}{\partial x_{k}^{+}}, \end{array}$$

A 3 $$\begin{array}{rl} \frac{\partial (\rho^{+}E^{+})}{\partial t^{+}}+\frac{\partial (\rho^{+}u_{k}^{+}E^{+})}{\partial x_{k}^{+}} & =-(\gamma -1)Ma^{2}\frac{\partial (p^{+}u_{k}^{+})}{\partial x_{k}^{+}}+\frac{1}{\text{Re}}(\gamma -1)Ma^{2}\frac{\partial (\tau_{ki}^{+}u_{i}^{+})}{\partial x_{k}^{+}}\\ & \;+\frac{\tau }{\text{Re}\mathrm{Pr}}\frac{\partial }{\partial x_{k}^{+}}\left[\lambda^{+}\frac{\partial T}{\partial x_{k}^{+}}\right]-\frac{\tau }{\text{Re}\mathrm{Pr}}\frac{\partial }{\partial x_{k}^{+}}\left[\rho^{+}D^{+}\frac{\partial c}{\partial x_{k}^{+}}\right] \end{array}$$

A 4 $$\begin{array}{rl} \text{and}\;\frac{\partial (\rho^{+}c)}{\partial t^{+}}+\frac{\partial (\rho^{+}u_{k}^{+}c)}{\partial x_{k}^{+}} & ={\dot{w}}^{+}+\frac{1}{\text{Re}Sc}\frac{\partial }{\partial x_{k}^{+}}\left[\rho^{+}D^{+}\frac{\partial c}{\partial x_{k}^{+}}\right], \end{array}$$

where the non-dimensional quantities are defined as

A 5 $$\begin{array}{rl} x_{i}^{+} & =\frac{x_{i}}{L_{\mathrm{ref}}};\;u_{i}^{+}=\frac{u_{i}}{u_{\mathrm{ref}}};\;p^{+}=\frac{p}{\rho_{\mathrm{ref}}u_{\mathrm{ref}}^{2}};\;\tau_{ki}^{+}=\frac{\tau_{ki}}{\rho_{\mathrm{ref}}u_{\mathrm{ref}}^{2}};\\ E^{+} & =\frac{E}{C_{p}T_{0}};\;{\dot{w}}^{+}=\frac{\dot{w}L_{\mathrm{ref}}}{\rho_{\mathrm{ref}}u_{\mathrm{ref}}};\;\rho^{+}=\frac{\rho }{\rho_{\mathrm{ref}}};\;\lambda^{+}=\frac{\lambda }{\lambda_{\mathrm{ref}}};\\ D^{+} & =\frac{D}{D_{\mathrm{ref}}};\;T^{+}=\frac{T-T_{0}}{T_{ad}-T_{0}}, \end{array}$$

where $E=C_{v}T+u_{k}u_{k}/2+H(1-c)$ is the specific internal energy and *H* is the heat of reaction per unit mass of reactants consumed. Therefore, the non-dimensional specific internal energy takes the following form:

A 6 $$E^{+}=\frac{1}{\gamma }(1+\tau T^{+})+\frac{1}{2}(\gamma -1)Ma^{2}u_{k}^{+}u_{k}^{+}+\tau (1-c).$$

In equations (A 1)–(A 4), $\text{Re}=\rho_{\mathrm{ref}}u_{\mathrm{ref}}L_{\mathrm{ref}}\text{/}\mu_{\mathrm{ref}}$ is the nominal Reynolds number, *Ma* = *u*_ref_/*a*_ref_ is the Mach number, $\gamma =C_{p}/C_{v}$ is the ratio of specific heats, Pr is the Prandtl number and Sc=Pr⋅Le is the Schmidt number with $\rho_{\mathrm{ref}}$, $\lambda_{\mathrm{ref}}$, $D_{\mathrm{ref}}$, $u_{\mathrm{ref}}$, $L_{\mathrm{ref}}$, $a_{\mathrm{ref}}$ and $\mu_{\mathrm{ref}}$ being the reference values of density, thermal conductivity, mass diffusivity, velocity scale, length scale, acoustic velocity and viscosity, respectively. For this analysis, the density, thermal conductivity, mass diffusivity, viscosity and acoustic speed of the unburned gas are taken to be $\rho_{\mathrm{ref}}$, $\lambda_{\mathrm{ref}}$, $D_{\mathrm{ref}}$, $\mu_{\mathrm{ref}}$ and $a_{\mathrm{ref}}$, respectively, while *S*_L_ and $10\delta_{\mathrm{th}}$ are considered to be *u*_ref_ and *L*_ref_, respectively. Standard values are assumed for Pr and $\gamma =C_{p}/C_{v}$ (i.e. Pr=0.7 and γ=1.4). The ideal gas law *P* = *ρRT* is considered, which takes the following non-dimensional form:

A 7 $$P^{+}=\frac{1}{\gamma Ma^{2}}\rho^{+}(1+\tau T^{+}).$$

Equations (A 1)–(A 4) are solved in conjunction with equation (A 7) in SENGA [11–17].
